# Pupillary Block and Secondary Iridocorneal Angle Closure Due to Posterior Chamber Air Following Deep Anterior Lamellar Keratoplasty

**DOI:** 10.22336/rjo.2026.23

**Published:** 2026

**Authors:** Aysun Sanal Dogan, Damla Nur Dinc, Hilal Toprak Tellioğlu

**Affiliations:** 1 Department of Ophthalmology, Saglik Bilimleri University, Diskapi Yildirim Beyazit Training and Research Hospital, Ankara, Turkey

**Keywords:** DALK, air bubble, pupillary block, angle closure, DALK = Deep anterior lamellar keratoplasty, IOP = Intraocular pressure, DM = Descemet’s membrane, BB = Big bubble, DL = Dua’s layer, BCVA = Best corrected visual acuity, K1 = Mean flat keratometry, K2 = Mean steep keratometry, Kmax = Maximum keratometry, AS-OCT = Anterior segment optical coherence tomography

## Abstract

**Background:**

To describe the mechanism and non-invasive management of pupillary block and secondary angle closure caused by posterior migration of an anterior chamber air bubble following deep anterior lamellar keratoplasty (DALK).

Material and method: A case report.

**Case presentation:**

A 27-year-old male with advanced keratoconus underwent DALK. On postoperative day 1, he developed pupillary block and secondary angle closure due to an air bubble that had migrated from the anterior to the posterior chamber. Intraocular pressure (IOP) was elevated, and the anterior chamber was shallow. Conservative treatment with mydriatics, systemic IOP-lowering agents, and head elevation led to rapid improvement. No surgical intervention was needed, and the graft remained clear, with a normal IOP, during follow-up.

**Discussion:**

Pupillary block caused by intracameral air has been reported mainly after endothelial keratoplasty procedures, whereas it is extremely rare following DALK. Unlike previously reported DALK cases that were associated with reverse pupillary block mechanisms, the present case involved posterior chamber migration of an anterior chamber air bubble, resulting in secondary angle closure.

**Conclusions:**

Although rare, pupillary block and secondary angle closure caused by an anterior chamber air bubble after DALK may occur. Early intervention with medical treatment and simple postural adjustment can help avoid surgical intervention.

## Introduction

**Fig. 1:**
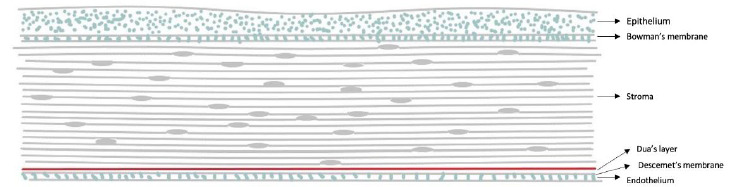
Anatomical layers of the cornea

Deep anterior lamellar keratoplasty (DALK) is a preferred option, particularly for stromal corneal diseases such as keratoconus, as it preserves healthy endothelium and lowers the risk of graft rejection [1]. In recent decades, various DALK techniques have been developed to expose Descemet’s membrane (DM) and the endothelium, or to leave minimal residual stroma, to improve visual outcomes [2]. Among various DALK techniques, the most commonly used method to achieve DM exposure is the big bubble (BB) technique, first described in 2002 [3]. This led to further exploration of the posterior corneal anatomy. In 2013, a new anatomical layer — Dua’s layer (DL) (**Fig. 1**)— between the deep stroma and DM was identified [4]. This discovery clarified the mechanics of type 1 and type 2 BB formation. In type 1 BB, the cleavage occurs between the posterior stroma and DL, leaving DL attached to DM and endothelium. This results in a more stable posterior surface, allowing effective graft adhesion with minimal air. In contrast, type 2 BB separates DL from DM, exposing only the bare DM, which is more fragile and may lead to difficulties in graft apposition [2]. Due to the fragile nature of the exposed tissue, particularly in type 2 BB procedures, more air may be required to ensure graft apposition. However, unintended redistribution of air within the anterior segment may increase the risk of postoperative complications such as pupillary block or angle closure [5]. Although rare, such complications can have significant clinical consequences. Our case highlights a rare but important postoperative complication—pupillary block and secondary angle closure—associated with type 2 BB and an anterior chamber air bubble.

## Case presentation

**Fig. 2:**
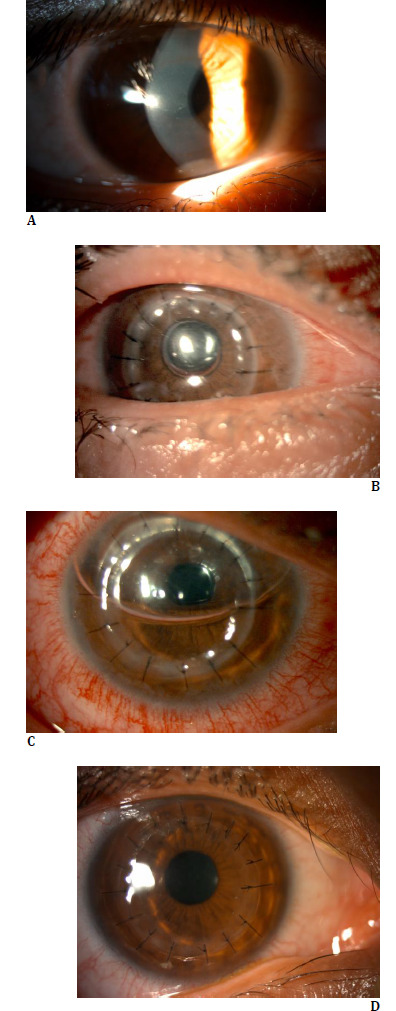
*Anterior segment images of the patient*. ***A*.** Preoperative examination showing prominent corneal nerves and apical steepening; ***B*.** On postoperative day 1, a flat anterior chamber was observed with an air bubble at the pupillary plane, accompanied by ciliary injection and anterior bowing of the iris; ***C*.** After intervention, air re-entered the anterior chamber, and iridocorneal touch began to resolve, initially in the superior quadrant; ***D*.** The graft became transparent, with normalization anterior chamber depth and resolution of ciliary injection

**Fig. 3:**
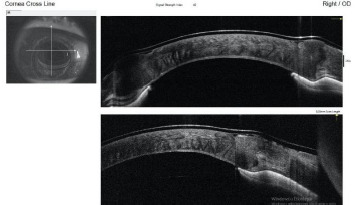
*AS-OCT images of the patient* showing peripheral iridocorneal touch and angle closure

A 27-year-old man presented with right-sided decreased vision and ocular misalignment. There was no systemic disease. On ocular examination, his best corrected visual acuity (BCVA) was counting fingers in the right eye and 20/25 in the left eye. There was sensory right exotropia in the strabismus examination. There were prominent corneal nerves and steepening at the apex in anterior segment examination (**Fig. 2A**). Fundus examination was normal. Given the clinical suspicion of keratoconus, corneal topography was performed. Corneal tomography (Sirius, CSO, Florence, Italy) revealed that the thinnest point of the right cornea was 325 µm, with a mean flat keratometry (K1) of 67.32 D, a mean steep keratometry (K2) of 69.32 D, and a maximum keratometry (Kmax) of 82.45 D. The patient underwent DALK due to advanced keratoconus. A 7.50 mm trephination was performed on the recipient cornea, and a 7.75 mm trephine was used to prepare the donor tissue. The BB technique was used during the surgery to separate the corneal stroma from Descemet’s membrane. Following type 2 BB formation, mechanical dissection and stroma removal were performed without complications. The donor graft was positioned and secured with 16 interrupted 10-0 nylon sutures. Additionally, an air bubble was injected into the anterior chamber to assist in graft apposition and alignment, and cyclopentolate drops were used for pupil dilation. The procedure was completed uneventfully, and the eye was patched with a therapeutic contact lens at the end. The patient was positioned supine with the chin up following the procedure. On postoperative day 1, the air bubble was visible only at the pupillary aperture, and the anterior chamber was flat. Slit-lamp examination revealed ciliary injection and anterior bowing of the iris (**Fig. 2B**). Intraocular pressure (IOP), assessed digitally due to the presence of a bandage contact lens, was found to be highly elevated. Anterior Segment Optical Coherence Tomography (RTVue-XR, Optovue Inc., Fremont, CA) demonstrated peripheral iridocorneal touch and angle closure (**Fig. 3**). Immediate treatment was initiated, including oral acetazolamide, intravenous mannitol infusion, 45-degree head elevation in the supine position, intensive topical corticosteroids to prevent donor corneal damage, and mydriatic drops (phenylephrine, cyclopentolate, tropicamide). Following these interventions, air entered the anterior chamber, and clinical signs gradually subsided over the following hours (**Fig. 2C**). The iridocorneal touch began to resolve, initially in the superior quadrant and subsequently in all quadrants. On postoperative day 3, IOP was 19 mmHg in the right eye and 16 mmHg in the left. The graft was transparent, with resolution of ciliary injection and restoration of normal anterior chamber depth and configuration (**Fig. 2D**).

## Discussion

In the literature, early postoperative IOP elevation due to pupillary block caused by an air bubble in the anterior chamber has been well described following lamellar keratoplasty [6]. In a series of eyes undergoing DALK, elevated IOP due to this mechanism was reported in 3 out of 44 cases (6.8%) [7]. As experience accumulated over the years, elevated IOP secondary to air in the posterior chamber and angle-closure reports arose regarding DMEK and DSEAK [8-10]. While some patients responded to medical therapy, others required surgical interventions such as pars plana decompression, iridotomy, or viscodissection [10]. However, a few cases of reverse pupillary block and secondary angle closure were reported in DALK surgery, all associated with free DM in the anterior chamber [5,11]. In the case described by Jabbour et al. [5], DM detachment led to the formation of a double anterior chamber, which obstructed the pupil and caused secondary angle closure. This required removal of the graft and dissection of residual stroma to evacuate the trapped air. Another report described a reverse pupillary block due to displaced host DM and residual stroma after DALK, which also resulted in angle closure. Initial medical management with timolol and acetazolamide was unsuccessful, and the patient underwent surgical decompression with a 26-gauge needle directed at the BB [1**1**]. Both cases involved reverse pupillary block mechanisms and required invasive intervention. In contrast, in our case, the air bubble migrated from the anterior chamber into the posterior chamber, pushing the iris forward and causing pupillary block. This led to secondary angle closure and a sudden rise in IOP. To our knowledge, there are no previously reported cases in the literature of pupillary block secondary to an anterior chamber air bubble following DALK, as observed in our case. Several factors may have contributed to the unusual migration of the air bubble toward the pupillary aperture in our case. The first factor may be related to the volume of air used. In type 2 BB, the bare DM is more fragile and lacks stromal support, which may require a larger air bubble to ensure graft adherence. In our case, a 0.25 mm oversized donor graft was used to stabilize better the central steepening typical of advanced keratoconus [12]. Although not routinely performed in DALK, an air bubble was intentionally injected into the anterior chamber to support the graft. Additionally, intraoperative mydriasis may have facilitated the passage of air toward the pupillary plane. Although the patient was instructed to maintain a strict supine position, even slight noncompliance or a minor head movement may have shifted the air bubble toward the pupil, causing pupillary block. Beyond the rarity of the mechanism, the case also highlighted the importance of early detection and non-invasive management in preventing long-term damage. The complication was promptly recognized on the 1st postoperative day, and immediate treatment was initiated, including topical mydriatics, IOP-lowering agents, and head elevation. These strategies facilitated redistribution of air into the anterior chamber, leading to rapid clinical improvement. Delayed recognition might have necessitated an invasive procedure, such as posterior air evacuation through a pars plana approach. Fortunately, no graft or glaucomatous damage was observed, highlighting the importance of early recognition and timely intervention in such cases.

## Conclusion

At the 3-year postoperative follow-up, the patient achieved a BCVA of 20/25 in the right eye, with a clear corneal graft and normal IOP. This favorable long-term outcome was made possible by early recognition and effective non-invasive management of the complication. To our knowledge, this is the first reported case of pupillary block caused by posterior chamber migration of an anterior chamber air bubble following DALK. This case highlights the importance of being aware of this rare but significant complication. Close postoperative monitoring and timely conservative intervention can prevent the need for surgical procedures and help preserve both graft clarity and visual function.
